# Menstrual cycle among adolescents: girls’ awareness and influence of age at menarche and overweight

**DOI:** 10.1590/1984-0462/2022/40/2020494

**Published:** 2022-01-05

**Authors:** Patrícia Marques, Tiago Madeira, Augusta Gama

**Affiliations:** a Research Centre for Anthropology and Health, University of Coimbra, Coimbra, Portugal.

**Keywords:** Menarche, Menstrual cycle, Adolescent obesity, Adolescent health, Menarca, Ciclo menstrual, Obesidade pediátrica, Saúde do adolescente

## Abstract

**Objective:**

To characterize the menstrual cycle (regularity and menstrual flow length), the prevalence of dysmenorrhea and self-monitoring of the cycle in students from Lisbon region, and explore the effect of chronological age, age at menarche and body mass index (BMI) on menstrual disorders.

**Methods:**

This is a cross-sectional study with 848 girls aged 12–18 years. A questionnaire about the sociodemographic context and characteristics of the menstrual cycle, and weight and height measures were assessed. BMI was classified according to International Obesity Taskforce criteria. A descriptive analysis of the variables was made, and *Odds Ratios* (ORs) and 95% confidence intervals (95%CIs) were determined.

**Results:**

Mean age at menarche was 12.4 years and mean BMI was 22.0kg/m^2^. Among adolescents, 59% have regular menstrual cycle, 83% have menstrual flow length of ≤6 days. 88% suffered from dysmenorrhea, among which 8,7% declare absenteeism from school and 49% took pain medication, and 65% self-monitor their menstrual cycle. Higher maternal education was associated with a higher self-monitoring of menstrual cycle among the sample (OR 1.60; 95%CI 1.15–2.17). Girls with menarche <12 years-of-age are more likely to have menstrual flow length of >6 days (OR 1.73; 95%CI 1.19–2.51) and dysmenorrhea (OR 1.87; 95%CI 1.11–3.16) than those with menarche ≥12 years-of-age. No significant association between BMI and menstrual cycle variables was observed.

**Conclusions:**

The results suggest that menstrual disorders are frequent and may be associated with early menarche, but not with BMI. It is important to encourage self-monitoring of the menstrual cycle to detect menstrual disorders timely and promote health and well-being.

## Resumo

**Objetivo:**

Caracterizar o ciclo menstrual (regularidade e duração da menstruação), prevalência de dismenorreia e automonitoramento do ciclo em estudantes da região de Lisboa, e explorar o efeito da idade cronológica, idade de menarca e índice de massa corpórea (IMC) nos distúrbios menstruais.

**Métodos:**

Este é um estudo transversal com 848 meninas entre 12 e 18 anos. Foi aplicado um questionário sobre contexto sociodemográfico e características menstruais e feita a medição do peso e altura. O IMC foi classificado pelos critérios da International Obesity Taskforce. Fez-se uma análise descritiva das variáveis e determinaram-se Odds Ratio (p<0,005; IC95%).

**Resultados:**

A média da idade de menarca foi 12.4 anos e do IMC foi de 22.0kg/m2. Entre as jovens, 59% afirmaram ter ciclo menstrual regular, 83% menstruação com duração ≤6 dias, 88% sofrem de dismenorreia, entre as quais 8,7% faltaram à escola e 49% consumiram analgésicos, e 65% automonitoram o ciclo menstrual. Uma educação materna elevada está associada a maior monitorização do ciclo menstrual entre as jovens (OR 1,60; IC95% 1,15–2,17). Meninas com idade de menarca <12 anos têm maior chance de ter menstruação com duração >6 dias (OR 1,73; IC95% 1,19–2,51) e dismenorreia (OR 1,87; IC95% 1,11–3,16) do que aquelas com menarca ≥12 anos. Não se observou associação entre o IMC e as características menstruais.

**Conclusões:**

Os resultados sugerem que os distúrbios menstruais são frequentes nas adolescentes e podem estar associados com a menarca precoce, mas não com o IMC. É importante incentivar a automonitoramento do ciclo menstrual para detectar adequadamente as perturbações menstruais e promover a saúde e bem-estar.

## INTRODUCTION

Menarche and menstrual cycle are indicators of female fertility and health.^1^ Menstrual cycle irregularities and primary dysmenorrhea are among the most common female complaints.^2,3^ However during the early years after menarche, the duration of the menstrual cycle may be shorter than 20 days or longer than 45, whereas normal cycles range between 21–34 days.^4^ Longer menstrual cycles may be related with anovulation, which occurs as a consequence of the immaturity of the hypothalamic-pituitary-ovarian axis.^4^

Primary dysmenorrhea (further referred to as “dysmenorrhea”), defined as pain occurrence with menses with no obvious pelvic pathology at the onset of menstruation, is also more frequent before 20 years of age.^5^ As reviewed by et al.^5^, primary dysmenorrhea is related to uterine contraction and vasoconstriction, the substantial increase in prostaglandin F2α in the endometrium, in the late secretory phase, results in increased myometrial tone and large increase of uterine contraction. However, other factors related with inflammation and stimulation of pain may be involved. Menstrual pain has negative consequences on girls’ quality of life and may interfere with their day-to-day life, resulting, for instance, in school absenteeism.^3,6^ Menstrual irregularities or dysmenorrhea are often considered “normal” during adolescence, but both may have an organic cause whose diagnosis can be delayed due to a lack of awareness of the normal pattern of the menstrual cycle. Educating girls to be aware of their own menstrual cycle is important to early detect disturbances resulting from a health issue and improve their health status.^2,4,7^

Age at menarche seems to be associated with menstrual patterns. Girls who have their first menstruation earlier tend to have shorter and more irregular menstrual cycles, bleeding between cycles, and are at a higher risk of suffering from primary dysmenorrhea.^3,8^ Some studies observed that younger age at menarche is related to higher body mass index (BMI).^9,10,11,12^ This association is controversial; however, obesity during adolescence is associated with earlier menarche.^13^ A higher BMI is also associated with menstrual irregularities and primary dysmenorrhea.^14,15^

Portuguese studies show that average age at menarche decreased from around 15 years in the beginning of the 20^th^ century to between 12–13 years^16,17^, and that adolescent’ overweight/obesity prevalence ranges between 20–25%.^18,19^ However, data on menstrual patterns and how they affect Portuguese female adolescents’ lives is scarce.

To promote female reproductive and overall health and wellbeing is important to educate girls and their parents concerning the normal menstrual cycle characteristics.^1,4^

Therefore, this study aimed to characterize menstrual cycle patterns in adolescents, regarding menstrual cycle regularity, menstrual flow length, and menarche age, to determine the prevalence and impact of dysmenorrhea on school absenteeism, to analyze menstrual cycle self-monitoring, and to explore the effect of chronological age, age at menarche, and overweight on menstrual cycle disorders.

## METHOD

This is a cross-sectional study with schoolgirls from the Lisbon Metropolitan Area, Portugal, focusing on menstrual cycle characteristics, age at menarche, and BMI. A total of 912 girls were observed, although 64 of them were excluded for not having had their menarche at the time of data collection. The final sample comprised 848 girls.

Data collection was conducted on a convenience sample of five schools: three secondary-high schools at Sintra municipality observed between January and May 2012 and two secondary-high schools at Lisbon municipality observed between January and March 2017.

This study followed the ethical principles for research involving human subjects. Ethical approval was obtained from *Sistema de Monitorização de Inquéritos em Meio Escolar* (MIME), *Direção Geral de Educação, Ministério da Educação* (Portuguese Ministry of Education) to fulfil all the legal requirements at the time the survey took place. When data collection was conducted, the Portuguese Commission for Data Protection determined that analyses of these anonymized data were exempt from review. An information letter describing the study was sent to the invited schools. Information about the study objectives and procedures were given to each participant, and written informed consent was obtained from the participants’ legal guardians, or by themselves in case they were 18 years old or older. Confidentiality, anonymity, and non-transmissibility of data were assured.

Female students were asked to complete an anonymous self-administered questionnaire which included questions concerning health and well-being, lifestyle, and sociodemographic characteristics. Information on menstrual cycle was collected using the Menstrual Disorder of Teenagers (MDOT) questionnaire.^20^

Anthropometric measures were taken using standardized procedures. The height and weight of each participant wearing light clothes and without shoes was measured by a trained researcher. Height (cm) was measured using a portable Seca 217 stadiometer scale with an accuracy of 0.1cm and weight (kg) was measured with an electronic portable Seca 872 scale to the nearest 0.1kg.

For this study, the data used comprised menstrual cycle characteristics, age at menarche, BMI, and family socioeconomic factors. Chronological age (Age) was categorized into two groups: young teenagers (12–14 years of age) and teenagers (15–18 years of age). Age at menarche was obtained by recall method. Girls who already had their first menstruation were asked to indicate as accurately as possible the date of their menarche (year and month). When the month was missing, a correction factor of 0.5 was considered. Age at menarche was categorized into two groups: <12 years old and ≥12 years old.

Menstrual cycle was characterized within the following characteristics: menstrual cycle regularity (regular or irregular), menstrual flow length (≤6 or >6 days), dysmenorrhea (yes or no), impact of dysmenorrhea on school absenteeism (yes or no), and pain medication consumption for dysmenorrhea (yes or no). Girls were also asked if they usually register the first day of menstruation (yes or no) as a proxy for self-monitoring their cycle. Categories of menstrual cycle parameters have been described elsewhere.^4^

Family socioeconomic variables used were parental education categorized into two levels (≤9 and >9 years of education) and parental marital status categorized into two groups married (married=married/cohabiting or separated= divorced/separated/single/widowed).

BMI (kg/m^2^) was calculated and the nutritional status classified according to the specific cut-off points for age and gender proposed by the International Obesity Task Force (IOTF)^21,22^ into two overweight categories (yes=overweight or obesity, or no=normal weight or underweight).

Mean and standard deviation for quantitative variables and distribution by absolute and relative frequencies for categorical variables were calculated. T-tests were used to compare means of continuous variables, and the χ^2^ test was used to compare frequencies among nominal variables. Binary logistic regression analysis (*Odds Ratios* [OR] and respective 95% confidence interval [95%CI]) was used to assess the association between the mother’s educational level (independent variable) and marking the first day of menstruation (dependent variable: 0=no, 1=yes), and to assess association between age at menarche (independent variable) and the dependent variables: menstrual cycle irregularity (0=no, 1=yes), menstrual flow length (0=≤6 days, 1=>6 days), and dysmenorrhea (0=no, 1=yes). In the latter, <12 years was used as reference group. A crude model and two adjusted models (using age, and age+BMI, respectively, as confounders) were developed. For the values calculated in this paper, p<0.05 was statistically significant at a 95%CI. Statistical analysis was performed using IBM through the *Statistical Package for the Social Sciences* (SPSS), Windows, version 24.0 (Armonk, NY: IBM Corp.).

## RESULTS

The final sample analyzed included 848 girls (Sintra, n=494; Lisbon, n=314) aged 12–18 years old. Mean age at menarche between the two surveys does not show significant difference (2012=12.4±1.2 years and 2017=12.3±1.3 years, respectively, p>0.05), and therefore, the two samples were analyzed together. An anthropometric evaluation of the participants was conducted, on average, 3.0±1.8 years after menarche.

Adolescents’ characteristics, regarding socioeconomic factors, weight status, and menstrual cycle are presented in Table 1. The girls’ mean age was 15.5±1.6 years old and 57.7% of them were 15 to 18 years old. Most of the girls were from families with mother and father with ≤9 years of education (61.8 and 60.0%, respectively) and parents were married (62.9%).

**Table 1.** Sociodemographic characteristics, weight status, and menstrual characteristics of adolescents.

Socioeconomic characteristics		n	%/mean±SD
Age (years)	Total	848	15.5±1.6
Age categories (years)	12–14	359	42.3
	15–18	489	57.7
Mother’s educational level	≤9 years	274	38.2
	>9 years	444	61.8
Father’s educational level	≤9 years	259	40.0
	>9 years	388	60.0
Parental marital status	Married	506	62.9
	Separated	298	37.1
Municipality	Lisbon	346	40.8
	Sintra	502	59.2
Weight status			
BMI (kg/m^2^)	Total	848	22.0±3.6
Overweight	Yes	204	24.1
	No	644	75.9
Menstrual characteristics			
Age at menarche (years)	Total	848	12.4±1.3
	<12	249	29.4
	≥12	599	70.6
Menstrual cycle regularity	Regular	410	58.7
	Irregular	288	41.3
Menstrual flow length (days)	≤6	694	82.8
	>6	144	17.2
Dysmenorrhea	Yes	747	88.3
	No	99	11.7
Pain Medicine consumption*	Yes	393	54.0
	No	335	46.0
School absenteeism*	Yes	65	8.7
	No	682	91.3
Mark the first day of the menstruation	Yes	545	65.0
	No	294	35.0

*Pain medicine consumption and school absenteeism were assessed only among females that reported dysmenorrhea (n=747); SD: standard deviation; BMI: body mass index.

The girls’ mean BMI was 22.0±3.6kg/m^2^ and the prevalence of overweight was 24.1%. The mean age at menarche was 12.4±1.3 years and 29.4% of girls were classified as having had their menarche at <12 years old.

Regarding menstrual cycles, 58.7% of the adolescents reported a regular cycle, 82.8% reported a menstrual flow length of ≤6 days. Dysmenorrhea was revealed by 88.3% of the adolescents. Among those girls, 54% stated using pain medication and 8.7% reported school absenteeism. Additionally, 65% of the adolescents usually register the first day of menstruation as a proxy for self-monitoring their menstrual cycle.

Table 2 provides the association between menstrual patterns and family socioeconomic factors, with self-monitoring of the menstrual cycle (“Mark the first day of menstruation”). The only significant association found was with maternal education (p=0.004). A higher percentage of girls who mark the first day of menstruation had a mother with >9 years of education (66%). Girls who have a mother whose education is >9 years are 1.60 (95%CI 1.15–2.17) times more likely to mark the first day of menses than those who have a mother whose education is ≤9 years (data not shown).

**Table 2.** Association between self-monitoring of menstrual cycle (“Marking the first day of menstruation”) with age, age at menarche, menstrual patterns, and family characteristics.

		Marking the first day of menstruation				
		Yes		No		p-value
		n	%	n	%	
Age (years)	12–14	217	61.8	134	38.2	0.106
	15–18	328	67.2	160	32.8	
Age at menarche	<12	151	61.1	96	27.7	0.134
	≥12	394	66.6	198	33.4	
Menstrual cycle regularity	Regular	280	68.8	127	31.2	0.219
	Irregular	184	64.3	102	35.7	
Menstrual flow length (days)	≤6	447	64.3	242	35.1	0.902
	>6	92	64.3	51	35.7	
Dysmenorrhea	Yes	485	65.6	254	34.4	0.238
	No	59	59.6	40	40.4	
School absenteeism	Yes	44	67.7	21	32.3	0.752
	No	500	64.7	273	35.3	
Mother’s educational level	≤9 years	162	59.1	112	40.9	0.004
	>9 years	308	69.7	134	30.3	
Father’s educational level	≤9 years	162	63.0	95	37.0	0.092
	>9 years	270	66.9	119	30.6	
Parental marital status	Married	215	42.9	286	57.1	0.340
	Separated	116	39.5	178	60.5	

Table 3 provides the association between menstrual characteristics and age, age at menarche, and overweight. Significant associations were observed between age and menstrual regularity (p<0.001) and menstrual flow length (p<0.001). Young teens report more irregular cycles (44.6 *vs*. 39.2%) and more days of bleeding (22.0 *vs*. 13.7%) when compared to teenagers.

**Table 3.** Association between menstrual characteristics and age, age at menarche, and overweight.

		Age (years)				p-value	Age at menarche (years)				p-value	Overweight				
		12 to 14 (n=359)		15 to 18 (n=489)			<12 (n=249)		≥12 (n=599)			Yes (n=644)		No (n=204)		p-value
		n	%	n	%		n	%	n	%		n	%	N	%	
Menstrual cycle regularity	Regular	149	55.4	261	60.8	<0.001	136	61.5	274	57.4	0.307	311	59.0	99	58.7	0.796
	Irregular	120	44.6	168	39.2		85	38.5	203	42.6		216	41.9	72	42.1	
Menstrual flow length (days)	≤6	273	78.0	421	86.3	<0.001	191	77.0	503	85.3	0.004	528	82.8	166	83.0	0.937
	>6	77	22.0	67	13.7		57	23.0	87	14.7		110	17.2	34	17.0	
Dysmenorrhea	Yes	304	85.2	443	90.6	0.180	230	92.4	517	86.6	0.017	565	87.9	182	89.7	0.490
	No	53	14.8	46	9.4		19	7.6	80	13.4		78	12.1	21	203	

Also, significant differences were found between age at menarche and menstrual flow length (0.004) and dysmenorrhea (p<0.017). Girls with earlier menarche (<12 years of age) more often reported longer menstrual flow length (23 *vs*. 14.7%) and suffering from dysmenorrhea (92.4 *vs*. 86.6%) than girls with menarche at age ≥12. No significant associations were observed between overweight and menstrual characteristics. However, overweight occurrence was higher among girls with menarche at <12 years of age when compared with those with menarche ≥12 years, respectively, 37.3 and 18.5% (p<0.001) (Data not shown in the table).

Three models were used to understand the effect of age at menarche on menstrual cycle characteristics and are presented in Table 4. Girls aged <12 years at menarche were 1.73 (95%CI 1.19–2.51) times more likely to have menstrual flow length of >6 days than those aged ≥12 years at menarche. Girls who had their menarche at age <12 were 1.87 (95%CI 1.11–3.16) times more likely to have dysmenorrhea than those at ≥12 years. After adjusting by age and age+BMI (models 2 and 3, respectively), the strength of the relationship remains significant.

**Table 4.** Logistic regression with menstrual disorders predicted by age at menarche <12 years.

Menstrual disorders	Model 1^(a)^		Model 2^(b)^		Model 3^(c)^	
	OR (95%CI)	p-value	OR (95%CI)	p-value	OR (95%CI)	p-value
Irregularity (n=698)	0.84 (0.61–1.17)	0.307	0.80 (0.58–1.12)	0.193	0.80 (0.57–1.13)	0.200
Menstrual flow > 6 days (n=838)	1.73 (1.19–2.51)	0.004	1.651 (1.11–2.41)	0.009	1.73 (1.18–2.55)	0.005
Dysmenorrhea (n=846)	1.873 (1.11–3.16)	0.019	1.96 (1.16–3.32)	0.012	1.94 (1.14–3.32)	0.015

^(a)^Crude Model; ^(b)^Adjusted for age (continuous); ^(c)^Adjusted for age and BMI (continuous); OR: *Odds Ratios*; 95%CI: 95% confidence interval.

## DISCUSSION

Overall, the results suggest that there is a significant association between early menarche (<12 years) and longer menstrual length (>6 days) and dysmenorrhea. There is no relation between menstrual characteristics and BMI.

Mean age at menarche was 12.4±1.3 years, which is in line with the mean values reported in Europe and North America, ranging from 12.5–13.5 years.^6,9,11,23,24^ It is also aligned with what has been reported for Portuguese adolescents in other studies.^9,17,25^ The percentage of early menarche determined was 29.4%, which is lower than that reported in a study with Portuguese girls (37.2%)^17^ and higher than the one from another study (26.6%).^25^ Additionally, 24.1% of the girls observed were overweight, which is in line with recent data from Portugal.^18^

In this study, it was observed that most of the girls have menstrual cycles according to the normal menstrual patterns.^4^ However, 41.3% of the girls reported irregular cycles and 17.2% reported menstrual flow length of >6 days. Irregular cycles were more prevalent among young teens (44.6%) than among teenagers (39.2%). Also, a higher percentage of younger teens reported longer menstrual flow (22.9%) compared to teenagers (13.7%). A Portuguese study with girls aged between 9 and 19 years reported that 23.8% of them had irregular cycles.^26^ Results from other European countries show that the prevalence of irregular cycles range from 8 to 9% and longer menstrual flow length is reported by around 19% of the females.^3,24^ These studies also showed that irregular cycles and longer menstrual flow length were more prevalent among young teens. Differences between our results and the ones by these studies may be related to different females age range, as well as other socioeconomic factors not addressed. Moreover, it was expected that young adolescents report having irregular cycles and longer menstrual flow days more frequently, considering that those disorders are frequent in the years after menarche and tend to normalize over time.^2,3^

About 88% of the observed girls suffer from dysmenorrhea, which is one of the most common complaints among adolescents.^27–29^ A lower prevalence of dysmenorrhea, ranging from 51–63%, was observed in other studies.^6,28^ Again, these differences may be related to the age of the sample.

This study’s results show that 8.7% of girls missed school due to dysmenorrhea. Menstrual pain is known as a factor with a negative impact on girls’ social life.^3,6^ Limitations in teenagers daily activities due to dysmenorrhea were observed in 65.7% of Portuguese adolescents/young adults in another study.^28^ An Italian study also observed that 12% of the young participants with severe dysmenorrhea were likely to declare absenteeism from school/work.^6^ However, when evaluating pain severity, individual factors such as personal pain perception should also be taken into account, as pain is a multidimensional phenomenon.^5^ Also, it is important to highlight that dysmenorrhea is influenced by several factors, ranging from health-related factors such as heavy menstrual flow, hormonal imbalance, to behaviors and habits such as smoking and alcohol consumption during contraceptive pill use, social aspects including education and religion, and obstetric factors, full-term delivery and breastfeeding, as described elsewhere.^14^

When accessing the self-monitoring of the menstrual cycle, it was observed that more than one-third of the girls do not register the first day of menstruation, which might be interpreted as an undervaluation of this important female event. Girls who keep track of their first day of menses were more likely to have a mother with >9 years of education. This suggests that education about menstrual cycle may start at home, in a mother-daughter context. It also highlights the importance of sociodemographic and familiar background, as well as cultural aspects, in sexual and reproductive health.^2,7^ Providing girls and parents the right knowledge about menstrual patterns is important to reduce the burden of menstrual disorders and diagnose potential pathologies that might affect their global and reproductive health.

Young teens showed a higher prevalence of overweight than teenagers (26.7 and 22.3%, respectively). A lower prevalence of overweight among older adolescents was also observed in other studies conducted in Portugal.^18,19^ A higher prevalence of overweight is associated with earlier menarche: 37.3% of the females who had their menarche before 12 years of age were overweight whereas only 18.5% of those who had their menarche after 12 were overweight. The relationship between BMI and age at menarche shows that lower age at menarche is related with higher BMI.^9–11^

Results show that menstrual disorders are associated with earlier age at menarche. The likelihood of having longer menstrual flow length (>6 days) and suffering from dysmenorrhea was higher among adolescents who had their menarche at age <12. The significant effect remained the same after adjusting the model for age, and age and BMI (age+BMI), meaning that the association between the variables remains the same regardless of age and/or BMI. This trend has been consistently observed in other studies.^3,8,30^

This study should be seen in the light of some limitations. Data were collected in a convenience sample, in the Lisbon Metropolitan Area, which compromises the representativeness of the results for other areas. However, the large size of the sample may reduce this limitation. This is a survey-based study and may be subject to information bias. Age at menarche was collected through recall methods and, therefore, the hypothesis of recall bias cannot be excluded; however, since menarche is a rather remarkable event in a female’s life, it is considered an appropriate method to assess age at menarche. Menstrual cycle and reproductive health are sensitive matters and, therefore, may be influenced by stigma and taboo, or even socially accepted responses, considering the age target of the study’s sample. Despite these limitations, this study is one of the first to analyze the relationship between menstrual cycle characteristics, age at menarche, and the girls’ self-monitoring of menstrual cycles in Portuguese teenagers. This study provides an up-to-date overview on menstrual cycle patterns in urban Portuguese adolescents. Menstrual disorders are common complains among adolescents. Early menarche and being overweight seem to increase the risk of suffering from those disorders. One-third of girls do not monitor their menstrual cycle, which is concerning as menstrual disorders may be caused by health issues that can be detected through the monitoring of one’s own menstrual patterns to detect alterations.

In conclusion, prevalence of menstrual disorders is high among adolescent girls. Early menarche seems to increase the likelihood of suffering from menstrual problems. One-third of the adolescents do not self-monitor their own menstrual cycle and the likelihood of this behavior increases for girls whose mothers have lower levels of education. More attention should be given to educate female adolescents to monitor their menstrual cycle and to understand what the normal menstrual patterns are, in order to seek timely healthcare when needed.

It is also important to identify the determinants of menstrual disorders and explore strategies to teach young girls to self-monitor their menstrual cycle, to promote health and well-being during this development phase.
